# “Two Minds Don’t Blink Alike”: The Attentional Blink Does Not Occur in a Joint Context

**DOI:** 10.3389/fpsyg.2018.01714

**Published:** 2018-09-12

**Authors:** Merryn D. Constable, Jay Pratt, Timothy N. Welsh

**Affiliations:** ^1^Faculty of Kinesiology and Physical Education, University of Toronto, Toronto, ON, Canada; ^2^Department of Psychology, University of Toronto, Toronto, ON, Canada; ^3^Centre for Motor Control, University of Toronto, Toronto, ON, Canada

**Keywords:** attentional blink, joint action, co-representation, joint information processing, cognition, attention

## Abstract

Typically, when two individuals perform a task together, each partner monitors the other partners’ responses and goals to ensure that the task is completed efficiently. This monitoring is thought to involve a co-representation of the joint goals and task, as well as a simulation of the partners’ performance. Evidence for such “co-representation” of goals and task, and “simulation” of responses has come from numerous visual attention studies in which two participants complete different components of the same task. In the present research, an adaptation of the attentional blink task was used to determine if co-representation could exert an influence over the associated attentional mechanisms. Participants completed a rapid serial visual presentation task in which they first identified a target letter (T1) and then detected the presence of the letter X (T2) presented one to seven letters after T1. In the individual condition, the participant identified T1 and then detected T2. In the joint condition, one participant identified T1 and the other participant detected T2. Across two experiments, an attentional blink (decreased accuracy in detecting T2 when presented three letters after T1) was observed in the individual condition, but not in joint conditions. A joint attentional blink may not emerge because the co-representation mechanisms that enable joint action exert a stronger influence at information processing stages that do not overlap with those that lead to the attentional blink.

## Introduction

In many daily tasks, such as cooking in a kitchen or searching for several items in a room, an individual will recruit the help of other people to complete the task more efficiently than if that individual performed the task alone. For this efficiency to occur, each individual in the group should know the overall goal of the task and the smaller sub-goals of their co-actors. Further, each individual should monitor their co-actors’ actions so that they can coordinate efforts and decrease redundant performance. Consider, for example, a situation in which Bob and Doug are searching for the items they need to go out and buy coffee and jelly doughnuts from the local coffee shop. Both Bob and Doug understand the super-ordinate goal of leaving the house efficiently and that, to achieve that goal, each person might be responsible for finding different items: Bob may be tasked with retrieving the wallet and the keys to the van while Doug must find the hats and mittens. To ensure the overall job is completed efficiently, Bob and Doug will likely maintain the goals of the other person in mind and monitor the performance of each other to know when the jobs are done. In this scenario, holding the co-actors’ task in mind will not only help to determine when the whole task is done, but it may also help to complete the overall task more efficiently because each individual would not ignore a target of their partner if they happen to come across it first: that is, Doug should not ignore and leave behind the keys if he finds them before Bob. Stopping their own search to identify and obtain the target of the partner might slow down their own sub-tasks, but may increase the efficiency of the overall search task. Thus, maintaining (co-representing) a partner’s goals in addition to one’s own goals may make the overall task more efficient despite a small and temporary cost of the individual’s own performance.

To gain an understanding of the processes enabling the completion of joint action and search tasks, researchers have typically adapted paradigms that have been developed to understand how people perform tasks individually to the joint action context for use with dyads (e.g., Sebanz et al., 2003; Welsh et al., 2005; Atmaca et al., 2008; Constable et al., 2015). The key feature of these studies is that the task is divided among two individuals such that each individual performs a sub-task that is essentially independent of their co-actor, but that collectively the pair of individuals are performing the full task in a social environment. The logic behind this approach is the following: If individuals working independently in this social environment do not co-represent or code for the actions and goals of the partner, then the behavioral effect that emerges when an individual completes the whole task while acting alone should not emerge in the performance of the co-actors. However, if individuals working independently in this social environment co-represent the actions and goals of the co-actor, then the behavioral effect that emerges when an individual completes the whole task while acting alone should emerge in the performance of the co-actors. The results of these joint action studies have been largely consistent with the latter hypothesis because behavioral effects that emerge when individuals complete a whole task alone also emerge in the behavior of individuals completing sub-components of the whole task. Thus, even though each individual has a distinct and independent task to complete, the data from joint action and search studies suggest that individuals know and code for the goals and tasks of their partner simultaneously to their own goals and responses.

An example of such a joint action and social search task that has been used to generate an understanding of the co-representation process is one in which two participants sit across from each other at a table and execute a series of movements from separate starting positions to a pair of target locations (e.g., Welsh et al., 2005, 2007, 2009; Hayes et al., 2010; Skarratt et al., 2010; Cole et al., 2012, 2018; Doneva and Cole, 2014; Janczyk et al., 2016; see also Ondobaka et al., 2012). In the studies by Welsh and colleagues, the targets appear randomly at one of two locations such that the location of the target on trial “*n*” does not predict the location of the target on trial “*n*+1.” Participants take turns responding to the targets in a paired-alternating manner such that the actor (Bob) would make two responses and then the partner (Doug) would execute two responses and so on (i.e., BBDDBBDD, etc.). With this method, the researchers were able to examine reaction times (RTs) on trials on which the target was in the same or a different location as the previous trial. When individuals perform such a sequence of responses, there are a multitude of studies that show RTs on trials in which the target is at the same location as the previous trial are longer than if the target is at a different location. These longer RTs for trials with repeated relative to different target locations are thought to emerge because shifting attention to and executing a response at one location eventually leads to the activation of an inhibitory code at that location. This inhibitory code hinders the return of attention and/or the reactivation of the response to that location – an inhibition of return (IOR) effect [e.g., Posner and Cohen, 1984; Maylor and Hockey, 1985; Welsh and Pratt, 2006; see Klein (2000) for review].

The key findings of the Welsh et al., (2005, 2007, 2009); studies [see also Cole et al. (2012, 2018)] was that an IOR effect emerged both when the participants acted two times in a row (an individual IOR effect on BB and DD trials) *and* when the participants acted after observing the response of their partner (a social IOR effect on BD and DB trials). Thus, IOR emerged when the individual executed their own response or observed the response of the partner. It is important to reemphasize here that, although both individuals executed movements to the same set of targets, their responses were independent from each other and were incidental to each partner’s task. In other words, the partner’s previous response did not predict nor was coordinated with the subsequent response of the actor, yet IOR emerged. Although some researchers have suggested that the social IOR effect emerges solely due to attentional mechanisms (see Atkinson et al., 2014; Doneva and Cole, 2014), the most common account is that the social IOR effect is generated because the knowledge and observation of the partners’ action lead to a co-representation and simulation of the partner’s response, subsequently activating the same mechanisms that generate the IOR effect when the person acts alone. In support of the hypothesis that the same mechanisms are activated following the execution and observation of the response, Welsh et al. (2009) found that the magnitude of the social IOR effects (RTs on same target trials minus RTs on different target trials) was significantly correlated with the magnitude of the IOR effect on individual trials. Overall, the data from the studies of the social IOR effect indicate that, even though two individuals complete independent tasks in succession in a common environment, the tasks, goals, and actions of the independent partners are co-represented and affect each other’s performance.

Similar co-representation and simulation accounts have been extended to account for other joint action and social search tasks such as the joint negative priming effect (Frischen et al., 2009; Welsh and McDougall, 2012). In these studies, participants are presented with a pair of displays (first a prime and then a probe display). Each display has a target and a distractor stimulus, and the task is to respond to the location of the target and ignore the location of the distractor. The location of the target and distractor varies from trial-to-trial and from prime to probe display. The two key trial types in the negative priming task are: (1) the baseline control trials – the target and distractor on the probe display appear at different locations from the target and distractor on the prime display and (2) the ignored repetition trials – the target on the probe display appears at the same location as the distractor on the prime display. It has repeatedly been demonstrated that RTs for probe targets on ignored repetition trials are longer than on baseline control trials. One of the predominant explanations of the longer RTs for probe targets of ignored repetition trials than on baseline trials is the selection inhibition account [Tipper, 1985; see Tipper (2001) for a review]. According to this account, selection of the target from the distractor on the prime display involves both the activation of the target information *and* the active inhibition of the distractor information. The inhibitory mechanism activated for the distractor on the prime display persists for some time. If the probe target is subsequently presented at the location of the prime distractor, the residual inhibition at that location hinders processing of the probe target at that location, increasing RTs. On baseline trials, the probe target is presented at a previously unoccupied location and thus processing of that probe target is unaffected by the selection process on the prime display and is relatively more efficient than the processing of the probe target on ignored repetition trials. Thus, this negative priming effect for probe targets occurs because of the successful target/distractor selection on the prime display.

In the individual version of the task used in studies of the joint negative priming effect (Frischen et al., 2009; Welsh and McDougall, 2012), a single participant completed the selection on both prime and probe displays. In the joint version, participants completed the task in pairs – one participant (Bob) completed the selection on the prime display and only responded to target 1, and the second participant (Doug) completed the selection on the probe display and only responded to target 2 (Frischen et al., 2009; Welsh and McDougall, 2012). These studies have revealed that, even though each individual is responsible for only responding to their own stimuli on separate displays (and could effectively ignore the stimuli in their partner’s display), a negative priming effect still emerges on joint trials – Doug’s RTs to target 2 on the probe trials are longer when target 2 in the probe display is presented at the same location as distractor 1 on the prime display than when target 2 is presented at a different location. This joint negative priming effect was suggested to emerge because, even though Bob’s (the first person) task precedes and is irrelevant to Doug (the second person) and Doug *could have* simply ignored the prime display, Doug will spontaneously co-represent the goals and actions of Bob and simulate Bob’s performance (i.e., simulate the target selection and response execution as well as the subsequent inhibition of the distractor). This co-representation and subsequent simulation of task performance activates the same mechanisms that would be activated if Doug worked alone and performed the entire task. This simulation leads to the same interference effects that emerge as though the individual performed the task on their own [see also Welsh et al. (2005) for a similar account of the social IOR effect]. In support of the hypothesis that the same mechanisms are activated on individual and joint trials, Welsh and McDougall (2012) reported that the magnitude of the negative priming effect on individual and joint trials was significantly correlated (see also Welsh et al., 2009). Overall, the results of the joint negative priming and social IOR studies provide evidence in favor of the hypothesis that co-actors maintain a representation of their partner’s task and may engage in a simulation of their partner’s performance when they observe that selection, even when it is temporally distinct and independent from their own task.

It is important to recognize that although the work reviewed here has shed some important new light on the processes of joint action and social searches, the tasks used in this work largely engage spatial and response selection processes (e.g., Sebanz et al., 2003; Welsh et al., 2005; Frischen et al., 2009; see also Ray and Welsh, 2011). That is, even though the social IOR and negative priming tasks have a temporal component in that participants consistently alternate their task performance, the main features that define these tasks are that participants must determine target from non-target locations and rapidly execute spatially defined responses to the selected target. How and if co-representation affects processes involving temporal selection and identification is largely unknown.

The primary goal of the present studies was to address this gap regarding temporal selection and identification by adapting a task that is better suited to investigating those processes in a joint action context: the attentional blink task. Importantly, the attentional blink is thought to result from the activation of mechanisms that are distinct from those that generate IOR and negative priming effects. In other words, the attentional blink task allows us to explore the co-representation of targets and temporal selection in joint action tasks in that participants alternate identifying targets in a task that does not involve spatial and response selection and execution as in previous joint tasks (i.e., we were not just measuring the IOR and NP processes in a different way).

In the typical (single participant) attentional blink task, an individual participant watches a series of stimuli (often letters) presented in rapid succession. The task of the participant is to watch the string of stimuli and determine if two targets are presented in the series of stimuli (e.g., Raymond et al., 1992, 1994). The key to the design of these tasks is that the two targets are embedded in the series of stimuli at different intervals apart from each other – the second stimulus could be presented immediately after the first target (Lag 1) or anywhere from 2 or more stimuli after the first target (Lag 2, Lag 3, etc.). The key finding from this work is that the detection of the second target (T2) is impaired by detection of the first target (T1), with the greatest impairment in the performance occurring when the T2 is presented two to three stimuli (Lag 2–3 or approximately 180 ms) after T1. Performance at identifying the T2 typically increases and returns to baseline levels when T2 is four or more stimuli after T1 (Lag 4+). This short-term decrement in performance for identifying the T2 at Lag 2–3 is known as the attentional blink [Raymond et al., 1992; see Dux and Marois (2009) for a review]. Although there is no single account of attentional blink effect that can explain all the findings, most accounts are based on the notion that the effect occurs because of early attentional mechanisms or limited loading or processing resources in working memory, not response selection and production processing (see Dux and Marois, 2009; cf. Jolicoeur, 1998).

Participants in the present studies completed a series of attentional blink tasks. Each task consisted of a series of rapidly presented letters and participants were required to determine if two targets appeared in the string of letters. The three conditions were: (1) an individual condition in which one participant responded to both targets, (2) a joint condition in which one person (Bob) identified the first target (T1) and the partner (Doug) identified the second target (T2), and (3) a second joint condition in which the roles were reversed – the partner (Doug) responded to T1 and the other person (Bob) responded to T2. The tasks were completed such that one joint task was always completed first with Bob responding to T1 and Doug responding to T2. After the first joint task, the participants completed their individual task conditions. For the final block, participants completed the joint task again but with the roles switched – Doug responded to T1 and Bob responded to T2. The rationale for choosing this specific order will be discussed in subsequent paragraphs.

The most theoretically relevant conditions for the present study were the joint conditions in which one of the participants responded to T2 only. The performance of participants on identifying T2 when their partner identified T1 (joint condition) provided an index of the joint attentional blink. If knowledge and co-representation of a co-actor’s task influences the mechanisms associated with the joint attentional blink, then a joint attentional blink will emerge. Such a finding would be consistent with the studies suggesting that knowledge and co-representation may lead to other social attention effects such as social IOR (Welsh et al., 2005) and negative priming (Welsh and McDougall, 2012). A joint attentional blink effect should emerge if the partner responding to T2 co-represents and simulates the performance of their partner who identifies T1. If knowledge and co-representation of the other persons’ task does not occur or if co-representation does not influence the processing of target information at these stages, then a joint attentional blink should not emerge.

Although the two joint conditions were the most critical, the individual condition served two important purposes. First, it served as a measure of internal validity to ensure that the stimulus conditions employed in the present study could evoke the attentional blink. Second, because each participant completed the individual task in between the two joint tasks, the individual task provided one-half of the participants with task experience prior to the critical joint task in which they identified T2 after their partner identified T1. Research has revealed that recent task experience can modulate the perception and imagination of action (e.g., Chandrasekharan et al., 2012; Wong et al., 2013) – two processes thought to involve action simulation. It is likely that task performance enhances these processes because experience strengthens the representations of the action and perceptual codes associated with the task, and leads to increased knowledge of the task and response conditions. Thus, providing one-half of the participants with task experience prior to responding to T2 allowed us to investigate whether or not experience with the task potentiates the co-representation and the subsequent joint attentional blink.

## Experiment 1

We adapted a conventional attentional blink task such that pairs of participants could complete both an individual attentional blink task and a joint attentional blink task. In the individual task, the participant identified both T1 and T2. In the joint task, one participant identified T1 and the other participant identified T2.

### Materials and Methods

#### Participants

Twenty-six undergraduate students from the University of Toronto participated in the experiment for course credit. Participants were aged 17–28 years (*M* = 19.88, *SD* = 2.78) and 19 were female. All participants had normal or corrected-to-normal vision. All participants provided informed consent prior to completing the tasks. The methods employed were approved by the Office of Research Ethics at the University of Toronto.

#### Apparatus

Stimuli were presented on a 1024× 768 CRT monitor with a refresh rate of 85 Hz. Presentation of the stimuli was controlled by Python using Psychopy (Peirce, 2007). All responses were entered on a standard QWERTY keyboard. The computer screen and keyboard were positioned on a table in front of the participants. During the individual block, participants sat directly in front of a computer screen (a distance of approximately 57 cm away). During the joint blocks, the participants sat side-by-side approximately 57 cm from the computer screen. The computer used in the joint tasks was different from that used in the individual tasks. The three computers were separated by an office partition. The position of the participants in the room and the task order they performed was randomized.

#### Design and Procedure

In each testing session, there was a total of four blocks of 240 trials. Each participant, however, only participated in three of the four blocks. The first block was always a joint condition, the second and third blocks were individual conditions that participants completed separately and simultaneously, and the last block was a joint condition. Specifically, the first block was a joint task in which Participant A responded to T1 and Participant B responded to T2. The second/third blocks consisted of individual task trials in which both participants completed the task individually by responding to both T1 and T2. The individual tasks were completed at the same time on separate computers. The final block of trials was a joint task trials in which Participant B responded toT1 and Participant A responded to T2.

**FIGURE 1:**
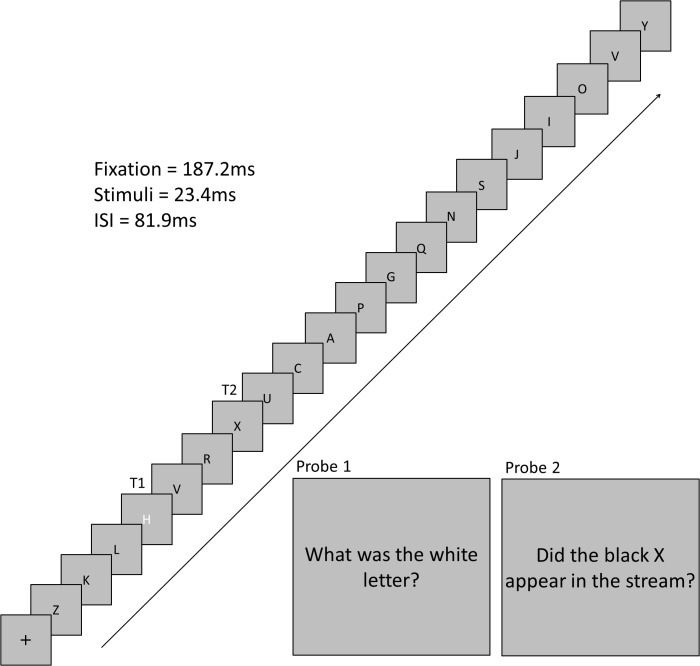
Time-course of a trial. This trial depicts a trial with a three stimulus lag between T1 and T2.

The experimental program, and hence the trial sequence, was the same for each condition. A trial began with a black central fixation cross that was presented on a gray background for 16 frames (187.2 ms). This cross was followed by a stream of 19 black letters and 1 white letter (1 VA). Each letter was presented for two frames (23.4 ms) with an inter-stimulus interval of seven frames (81.9 ms). Each non-target letter was selected from a pool of letters without replacement. T1 was selected from a pool of eight target letters, was colored white, and could appear at position 4, 5, 6, or 7 in the letter stream. A T1 was presented on every trial. T2 was always a black X and could appear 1, 3, 5, or 7 letter positions after T1. T2 was presented on 50% of trials. Participants were instructed to remember T1 and T2 to respond to two probe questions following the stream of letters (**Figure 1**) using the keyboard. For T1, participants pressed the key that corresponded to the identity of the letter. For T2, participants pressed “Y” or “N” to indicate if they detected the presence of the back “X’ or not, respectively. The response for T1 was always inputted prior to the response for T2.

Trials in the different task conditions were always the same. For the individual condition, participants identified and responded to both T1 and T2. Participants shared the task in the joint blocks – one participant would respond to T1 and the other responded to T2. For a given block of trials in the joint conditions, the role of the participants remained the same such that one participant (Bob) responded to T1 and the other participant (Doug) responded to T2 in the first joint task, and then changed roles in the second joint task block so that Doug responded to T1 and Bob responded to T2 in the last block of trials. Although each participant was present for the instructions and knew the task of their partner, they were not specifically instructed to attend to or monitor their partner’s task. In between the two joint tasks, each participant completed an individual block in which one person responded to both T1 and T2.

### Results and Discussion

**FIGURE 2:**
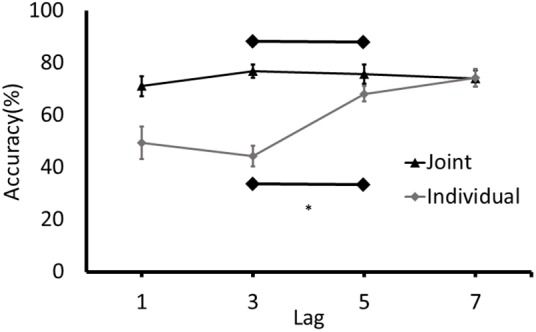
Detection rates of T2 (% of correctly identified as a function of the targets presented) for Experiment 1. Error bars represent standard error of the mean. ^∗^Denotes significance at p < 0.05.

Accuracy rates for T2 at each lag were calculated. For individual blocks, responses at T2 were only analyzed if the response at T1 was accurate. For the joint blocks, responses at T2 were analyzed regardless of accuracy at T1 because responses were made by two separate individuals (cognitive systems) and participants were not given any specific instructions to monitor the performance of their partner on T1. T1 was identified accurately on an average of 95.61% trials (*SD* = 4.70%) on the joint task and an average of 90.46% trials (*SD* = 7.49%) in the individual task. Data sets characterized by exceptionally low (below 50%) T2 accuracy at Lag 7 (at a time point in which identification should be at baseline levels; i.e., high) were removed prior to the analysis. This performance criterion accounted for the removal of two paired data sets in the joint condition and six individual data sets. To determine if an attentional blink was present in each condition, the analysis focused on the difference between the accuracy of detecting T2 at Lag 3 and Lag 5 (MacLean and Arnell, 2012). Separate paired samples *t*-tests were conducted on the individual and joint conditions (**Figure 2**).

An attentional blink was detected in the individual condition with T2 detection rates at Lag 3 being lower than at Lag 5, *t*(19) = -7.14, *p* < 0.001, 95% CI of Lag 3/Lag 5 difference [-30.84, -16.86]. Conversely, no joint attentional blink was observed, *t*(21) = 0.552, *p* = 0.587, 95% CI of Lag 3/Lag 5 difference [-3.36, 5.79]. To further determine if participants demonstrated an attentional blink in the joint task with a magnitude that is consistent with the attentional blink in the individual task, the difference between the detection rates at Lag 3 and Lag 5 in the joint task was calculated for each participant and compared to the 95% confidence intervals for the attentional blink in the individual task (-30.84 to -16.86). Only 1 of the 22 participants had a Lag 3/5 difference in the joint task that was in the range of the difference scores in the individual task.

To further explore the possibility that an attentional blink was present in the individual and joint conditions, the detection rates for T2 at Lags 3 and 5 in the different tasks were submitted to separate Bayesian analyses. This analysis has the benefit of generating an estimate of the amount of evidence in favor of the alternative hypothesis that there is an attentional blink and the null hypothesis that there is no attentional blink in the different conditions. The model used in the Bayesian analysis specified that the detection rates in the Lag 5 condition would be higher than the Lag 3 condition. The results of the analysis were consistent with results of the *t*-tests. That is, the estimated Bayes factor (BF) for the individual condition indicated that the data were 40,789 times more likely under the alternative hypothesis than the null hypothesis (BF_10_ = 40,789). This BF equates to extreme evidence in favor of the alternative hypothesis that there was an attentional blink in the individual condition. For the joint condition, the BF indicated that the data were 6.462 more likely under the null hypothesis (BF_10_ = 0.155). This result is considered as a moderate evidence in favor of the null hypothesis that there would be no difference between the detection rates for the Lags 3 and 5 in the joint condition. Overall, the results of the *t*-tests, Bayesian analyses, and the confidence intervals of the difference in detection rates at Lags 3 and 5 are consistent and provide converging evidence for the conclusion that an attentional blink was present in the individual condition, whereas no attentional blink was present in the joint condition.

As discussed earlier, it could be that experience performing a task increases the knowledge of the task and increases the potential for, or strength of, the co-representation and simulation of the partner’s task. As such, a joint attentional blink might only emerge after the participant responding to T2 in the joint task has experience performing both parts of the task in the individual condition; that is, activation of the mechanisms leading a joint attentional blink for individual participants may be dependent on the person being able to form a representation of the whole task. To test this prediction, additional analyses were performed on the subgroup of participants who performed the individual task before they completed the joint task in which they responded to T2 – the group of participants who identified T2 in the last block of trials. No joint attentional blink was observed in this subgroup, *t*(10) = 1.07, *p* = 0.31, 95% CI of the Lag3/Lag5 difference [-10.27, 3.61]. The results of the Bayesian analysis that tested a model where detection rates were lower at Lag 3 than at Lag 5 revealed that the data were 1.27 times more likely under the null hypothesis (BF_10_ = 0.79). This analysis provides only anecdotal/inconclusive evidence in favor of the null hypothesis. Despite the low sample size in this case, it is clear that there is no behavioral evidence in favor of a joint attentional blink that, at an individual level, is a robust phenomenon.

## Experiment 2

Although an attentional blink was present in the individual task where the individual responded to both T1 and T2, there was no evidence for an attentional blink in the joint conditions of Experiment 1. This finding stands in contrast to previous joint visual search literature in which selection by the partner on the preceding trial/display subsequently effects the selection of the individual (e.g., Frischen et al., 2009; Welsh et al., 2005; Welsh and McDougall, 2012). Thus, it is possible that co-representation does not influence the mechanisms leading to the attentional blink. It is interesting to note, however, that there has been one previous report of a null joint effect – the psychological refractory period [see Dux and Marois (2009) for some discussion in the mechanisms involved in this effect]. Interestingly, Liepelt and Prinz (2011) reported that a social psychological refractory period was not spontaneously elicited in conditions similar to Experiment 1 in which no specific instructions were given to participants to monitor the partner’s performance. A social psychological refractory period was observed, however, when participants were instructed to “monitor” their partner’s task. These instructions essentially asked participants to perform the whole task as an individual, but only actually respond to one-half of the task.

In consideration of the results of the findings of Liepelt and Prinz (2011), a second experiment was conducted to determine if specific instructions to monitor the performance of the partners could produce a joint attentional blink. Specifically, in Experiment 1, participants were not given any specific instructions for the participants to monitor the performance of the partner and co-representation and the mechanisms of the attentional blink were left to spontaneously emerge. Thus, Experiment 2 was conducted to determine if a joint attentional blink would emerge when participants were specifically asked to monitor what their partner was doing.

### Materials and Methods

#### Participants

Forty-four undergraduate students from the University of Toronto participated in the experiment for course credit. A larger sample size was collected for Experiment 2 to increase the power for the analysis on the subgroup of participants who completed the individual task before completing the joint task – the subgroup that was analyzed to determine if completing the individual task first increases the potential for observing an attentional blink in the joint task. Participants were aged 18–30 years old (*M* = 18.78, *SD* = 1.88) and 26 were female. All participants had normal or corrected-to-normal vision.

#### Design, Stimuli, Apparatus, and Procedure

All aspects of Experiment 2 were identical to those of Experiment 1 except for two important differences. First, the experimenter specifically instructed participants to monitor the performance of their partner during the joint task. That is, participants were told that they would receive global feedback on their performance on the trials. Participants were also told that, to determine who made an error on an incorrect trial, they would need to pay attention to the other person’s task. Global feedback was provided to participants after the response to the T2 was registered. If both participants answered correctly, they were notified that they were correct. If one participant made an error or both participants answered incorrectly, then they were notified that they were incorrect. Note that this manipulation is only a subtle promotion of monitoring behavior because if participants had faith in their own answer and abilities, then they would not need to monitor what the other person was doing. Further, there was no direct incentive for participants to monitor because they were not asked if the other person made a correct response or not.

The second difference was that the number of trials in each block was decreased from 240 in Experiment 1 to 160 in Experiment 2. Because the proportions of target present and absent trials remained the same, this decrease in overall trial number meant that there were only 80 trials on which T2 was present in the given task. The number of trials was decreased in Experiment 2 because the global feedback took additional time to deliver. Thus, to maintain relative consistency in the overall time required to complete the task (and prevent boredom), the number of trials were decreased.

### Results

The data from one participant in both conditions were removed because they only completed half of the trials. One joint data set was lost along with five individual data sets because the program failed to record the output file correctly. The data from one final participant from the individual condition was removed because their accuracy rate for T1 was 0%. Accuracy rates for T2 at each lag for each participant were then calculated. Accuracy rates for T2 were calculated the same way as in Experiment 1. For the individual task, T2 accuracy was only considered for trials on which T1 was correctly identified, whereas T2 accuracy on all trials was considered for the joint task. T1 was identified accurately on an average of 93.68% trials (*SD* = 6.01%) on the individual task and an average of 97.07% trials (*SD* = 5.75%) in the joint task. All participants had accuracy rates for T2 above 50% at Lag 7 and, as such, all remaining data were retained.

**FIGURE 3:**
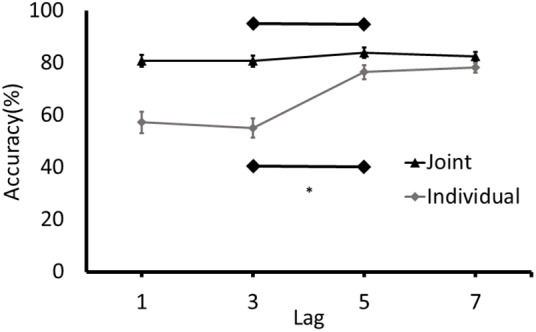
Detection rates of T2 (% of correctly identified as a function of the targets presented) for Experiment 2. Error bars represent standard error of the mean. ^∗^Denotes significance at p < 0.05.

Consistent with the approach to analysis in Experiment 1, separate paired samples *t*-tests and the equivalent Bayes test were conducted for joint and individual conditions on accuracy for T2 at Lag 3 and Lag 5. An attentional blink was detected in the individual condition, *t*(37) = -6.63, *p* < 0.001, 95% CI of Lag3/Lag5 differences [-28.06, -14.93]. The results of the Bayesian analysis are consistent with this finding: the data were 334,853 more likely under the alternative hypothesis, which is extreme support for a difference between Lag 3 and Lag 5 in the individual condition (BF_10_ = 334,853). Conversely, as can be seen in **Figure 3**, no attentional blink was observed in the joint condition, *t*(41) = -1.57, *p* = 0.12, 95% CI of difference scores [-7.252, 0.911]. The BF was unable to differentiate between support for the null and the alternative hypotheses (BF_10_ = 0.973). Finally, as in Experiment 1, the number of participants who demonstrated a joint attentional blink of the magnitude of the attentional blink in the individual task was determined by comparing the difference between the detection rates at Lag 3 and Lag 5 in the joint task to the 95% confidence intervals for the attentional blink in the individual task (-28.06 to -14.93). Only 8 of the 41 participants had a Lag 3/5 difference in the joint task that was in the range of the difference scores in the individual task.

Although completing the individual task before identifying T2 in the joint task did not seem to potentiate the joint attentional blink in Experiment 1, this analysis was conducted on a relatively low sample size. With the larger sample size in Experiment 2, we again conducted a paired sample *t*-test on the participants who performed the individual task before responding to T2 in the joint task – the group of participants who identified T2 in the last block of trials. Consistent with the findings of Experiment 1, no joint attentional blink was observed in this subgroup in Experiment 2, *t*(19) = -1.70, *p* = 0.11, 95% CI of the Lag3/Lag5 differences [-12.29, 1.29]. The results of the Bayesian analysis in which the detection rates at Lag 3 were compared to those at Lag 5 again provided inconclusive evidence that is slightly in favor of the alternative (BF_10_ = 1.47). Overall, even with the increased sample size and instructions that prompted participants to monitor the behavior of the partner, an attentional blink did not clearly emerge in the joint task.

### General Discussion

The purpose of the present study was to determine if an attentional blink would emerge in a task in which two people search for two different targets in a series of rapidly presented characters. Although robust attentional blinks emerged in the individual task in both Experiments 1 and 2 (accuracy at detecting T2 at Lag 3 was worse than at Lag 5), no such effect emerged in the joint task. Interestingly, neither previous experience with the task (i.e., completing the individual task prior to the joint task) nor instructions to monitor the performance of the person identifying T1 potentiated or activated the mechanisms of attentional blink in the joint task. Overall, the absence of the attentional blink in the joint task suggests that the mechanisms that generate the attentional blink were not activated when individuals were aware that their partner must identify the first target.

The finding that the detection of T2 was not affected in the joint task was unexpected given the joint action and social attention literature showing that individuals spontaneously co-represent and simulate the performance of their partner. In particular, in the studies of the joint negative priming effect (Frischen et al., 2009; Welsh and McDougall, 2012), the participant responding to the second (probe) display *could* completely ignore the first (prime) display because it is irrelevant to their task. Nonetheless, the joint negative priming effect emerged, suggesting that the participant responding on the probe display not only pays attention to the prime display, but also engages in the target/distractor selection process that leads to negative priming. Based on the findings of the joint negative priming effect (and similar findings in the social IOR effect; e.g., Welsh et al., 2005, 2007), it was predicted that the person responding to T2 could spontaneously co-represent their partners task and search for and identify T1 even though it was not part of their task. Evidently, such was not the case.

The absence of the joint attentional blink is similar to previous research on the attentional blink when individuals act alone. Specifically, Raymond et al. (1992) reported that the accuracy of responses to T2 was essentially unaffected in a task in which T1 was present, but the participant was instructed to ignore it. Thus, on first glance, it might not seem surprising that the detection of T2 in the joint task was not affected by T1 in the present studies because the participant detecting T2 did not ever have to identify and could effectively ignore T1. However, previous work that examined how the (non)identification of T1 affected the processing of T2 was always conducted in individual task contexts (i.e., without the presence of a co-actor identifying T1 and identifying T1 was not relevant at all). In the present study, each co-actor knew the task of their partner: the participant detecting T2 knew that the other participant was attempting to identify T1. Further, previous work using other social visual search tasks has revealed that the preceding action of a partner affects the performance of an individual in a manner that is similar to when the individual performs the entire task on their own, even if that response is independent of and not immediately relevant to the subsequent response (e.g., Welsh et al., 2005; Frischen et al., 2009; Welsh and McDougall, 2012). Thus, the absence of a social attentional blink requires a theoretical explanation, and a detailed discussion of the possible reasons why will be the focus of the remainder of the paper.

#### Co-representation

Previous information processing effects observed in joint contexts were suggested to emerge because co-actors observed and knew (co-represented) their partners’ task and response, and that this co-representation leads to the spontaneous activation of the mechanisms that are activated when the individual performs the whole task on their own (e.g., Sebanz et al., 2003; Welsh et al., 2005; Frischen et al., 2009). Based on this premise, it was predicted that each partner in the present studies would co-represent the task of the partner. As a result of this co-representation, even though they were not required to respond to T1, the participant responding to T2 alone would represent (and perhaps simulate) the task of the partner and that this co-representation would subsequently activate the mechanisms leading to the attentional blink. Such was evidently not the case. Before addressing why the effect did not emerge, two further observations will be discussed.

The first observation is that completing the individual task before the joint task did not affect the emergence of the joint attentional blink. Completing the individual task first could have increased the potential for a joint attentional blink because recent work suggests that experience with a movement task increases the accuracy of action perception (e.g., Chandrasekharan et al., 2012; Wong et al., 2013), increases the responsiveness of cortical areas activated during action observation (Calvo-Merino et al., 2005; Catmur et al., 2007), and affects the manner in which a co-actor adapts their actions for their partner (Ray et al., 2017). Previous experience is thought to have these effects because performance of the task (generating the action and sensing and perceiving the outcomes of the action) establishes, refines, and/or strengthens the coupling between the representations of the action and the perceptual consequences of those actions (Prinz, 1992; Hommel et al., 2001; Kunde, 2001; Elsner and Hommel, 2004; Gozli et al., 2016). Because it is these coupled perception-action codes that are thought to be activated during action observation and joint action, experience-based enhancements of these perception-action codes would have increased the knowledge and potential strength of the co-representation processes thereby increasing the potential for a joint attentional blink. No joint attentional blink, however, was observed in the performance of these individuals who gained task experience before performing the T2 detection in the joint task.

The second observation is that an attentional blink did not emerge even under instructions to monitor the performance of the partner (Experiment 2). These overt instructions to monitor the performance of the partner that identified T1 were expected to promote co-representation and the potential for the activation of the mechanisms that would generate a joint attentional blink. The absence of a joint effect under these instructions is not consistent with the findings in a paper reporting a social psychological refractory period effect – this effect only emerged under instructions that promoted partners to monitor each other’s performance (Liepelt and Prinz, 2011). However, it is possible that the social psychological refractory period effect emerged (though not spontaneously) because it involves response initiation or selection processes (Lien and Proctor, 2002) similar to many other effects that have companion joint effects such as the joint Simon effect (Sebanz et al., 2003) and the social IOR effect (Welsh et al., 2005).

So why was it that the attentional blink did not emerge in this study? First, despite instructions and expectations, it is possible that the participant responding to T2 did not know what the partner was doing and, as such, did not engage in co-representation. Without co-representation, joint effects are unlikely or unable to emerge. Although this possibility cannot be definitively ruled out, we believe it is likely that co-representation did occur because both participants were present during the delivery of the instructions and there is a wealth of previous research showing that joint effects (presumably due to spontaneous co-representation) under such conditions. Further, the participants in Experiment 2 were explicitly instructed to monitor the performance of the partner. Finally, the joint attentional blink did not emerge even in the subgroup who experienced the individual task prior to completing the T2 detection in the joint task – the subgroup who definitely had knowledge of both of the task components. Thus, we are confident that each participant knew the task and that co-representation occurred. The discussion will now turn to possible reasons why an attentional blink did not emerge despite co-representation.

#### Potential Reasons Why the Joint Attentional Blink Did Not Emerge

Based on the assumption that co-representation did occur, it seems that co-representation does not exert an effect upon the processes linked to the attentional blink. There are a number of possible reasons why the joint attentional blink did not emerge. The three most likely possible accounts will be addressed in turn. First, note that the majority of the previous studies on joint action have accounts that emphasize the role of “action” processing in generating the effects – processes that operate in spatial attention and response planning and selection such as the joint Simon effect (Sebanz et al., 2003), joint negative priming (Frischen et al., 2009; Welsh and McDougall, 2012), and social IOR (Welsh et al., 2005). Although some explanations of the attentional blink effect have a response selection component (Jolicoeur, 1998), the majority of the accounts of the attentional blink hold that the attentional blink emerges because of earlier attentional processes and/or limitations in the loading of or processing of information in working memory (see Dux and Marois, 2009). Hence, it is possible that mechanisms of co-representation preferentially operate on the level of decision making, response selection, and response programming rather than at earlier attentional and working memory processes.

In this context, it should be noted that there is evidence that the presence of another individual does affect perceptual and attentional processing. For example, there is evidence for spontaneous visuospatial perspective taking across a number of tasks (e.g., Böckler et al., 2011; Freundlieb et al., 2016, 2017, 2018). Further, Böckler et al. (2011) reported that the global/local processing of a stimulus was affected by the partner’s level processing (performance was less efficient when co-actors were to report a different level of feature than when they were to report the same level of feature). Finally, Constable et al. (2015) revealed that an object-specific recognition effect was altered by the hand posture of a co-actor. Interestingly, all these perceptual and attentional tasks, such as negative priming and IOR, involve a spatial dimension either regarding the features of the stimuli or of the co-actor. Thus, the attentional blink might not have emerged in the joint condition because the task employed in the present study is essentially non-spatial in nature (all stimuli were presented centrally), involved stimuli that were distinguished based on timing and identity, and did not involve response selection during the critical period of processing (cf. Dolk et al., 2014; Dittrich et al., 2017; for demonstrations of a spatial effect where co-representation may not exert an influence). In sum, the joint attentional blink might not have emerged because the processes activated and affected by co-representation and those involved in attention blink do not overlap.

Another, potentially related, possibility concerns the conceptual overlap (or non-overlap) in tasks. Much in the same way observing another person’s actions interfere more with one’s task when they are relevant for one’s own task (Bortoletto et al., 2013), perhaps another person’s task only interferes when there is close conceptual or dimensional overlap across tasks. For example, in the social Simon task (Sebanz et al., 2003), there is spatial and color features of the targets that are shared, or at least relevant, across participants. Similarly, in the work of Böckler et al. (2011) and Constable et al. (2015), perceptual state is relevant for the task. In the case of the present attentional blink task, the two tasks might not have had sufficient conceptual overlap to generate the joint effect – there is a temporal staggering of the stimuli: one partner completes an identification task before the other partner completes a detection task of a white stimulus in a string of black stimuli. These differences might have made the overall joint task less of a dynamic interaction than typical joint action tasks (e.g., Welsh et al., 2005; Frischen et al., 2009) and make each partner’s task more conceptually distinct.

A final explanation of the findings concerns the mental (attentional) states induced by completing a task with another individual. Previous work has revealed that if participants acting alone are required to complete an additional task (such as thinking about a holiday) while concurrently doing the attentional blink task, the attentional blink effect is attenuated (Olivers and Nieuwenhuis, 2005, 2006). Further, positive affect has also been shown to attenuate the attentional blink (Olivers and Nieuwenhuis, 2006). Given that positive affect is linked with diffusion of attention (Ashby et al., 1999), there is converging evidence suggesting that a diffuse attentional state can attenuate the attentional blink. Because of the social nature of the present joint task, it is also possible that the resulting positive environment and affect in the joint condition may have led to a diffuse attentional state. Thus, a joint attentional blink might not have been observed because of this diffuse attentional state. It should be noted, however, that previous studies typically report attenuated attentional blink effects rather than an abolishment of the effect as seen in the present study. As such, we feel that it is unlikely that a diffuse attentional state was the sole source of the absence of a joint attentional blink in the present study.

## Summary

In sum, the two experiments reported herein provide no evidence for the emergence of a joint attentional blink even when participants had previous task experience and specific instructions to monitor the performance of the partner. The possible reasons for the lack of such a joint effect are explored which can guide future research into understanding joint temporal processes.

## Ethics Statement

This study was carried out in accordance with the recommendations of the Declaration of Helsinki. The protocol was approved by the the Office of Research Ethics at the University of Toronto. All subjects gave written informed consent in accordance with the Declaration of Helsinki.

## Author Contributions

MC designed the study, ran the experiment, and edited the manuscript. TW wrote the manuscript. JP consulted on the design of the study and the manuscript.

## Conflict of Interest Statement

The authors declare that the research was conducted in the absence of any commercial or financial relationships that could be construed as a potential conflict of interest.
